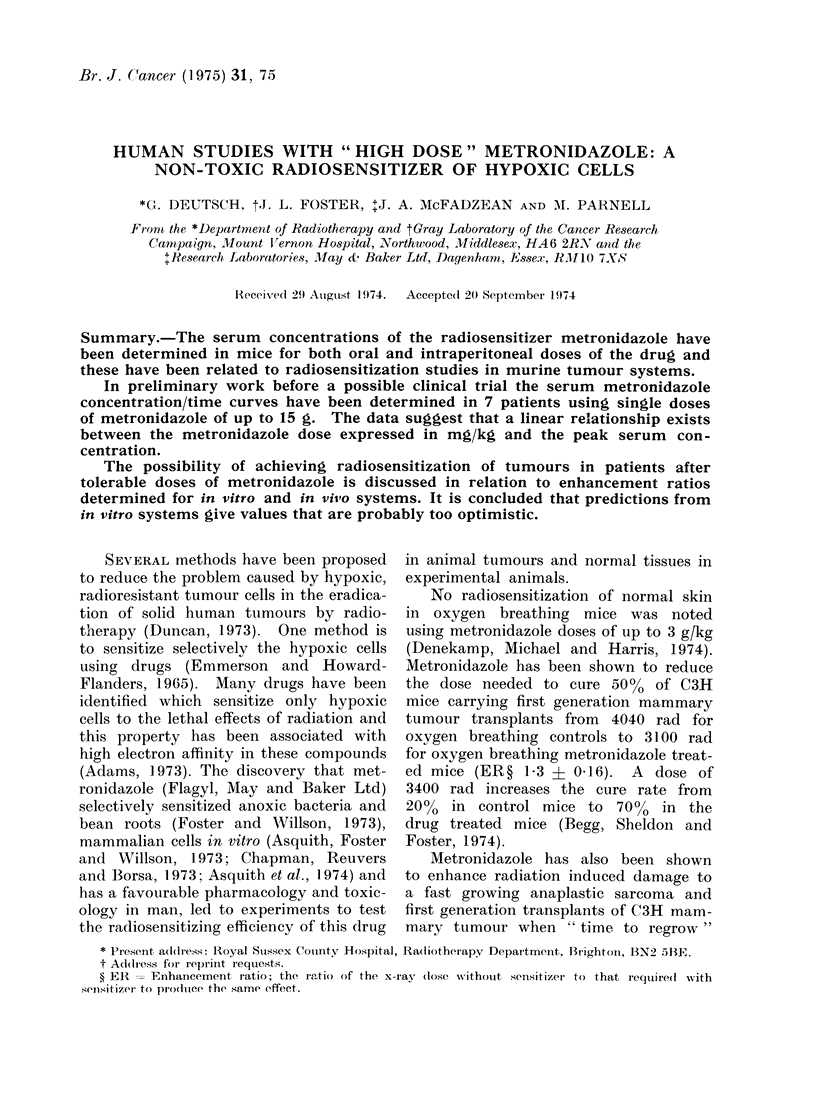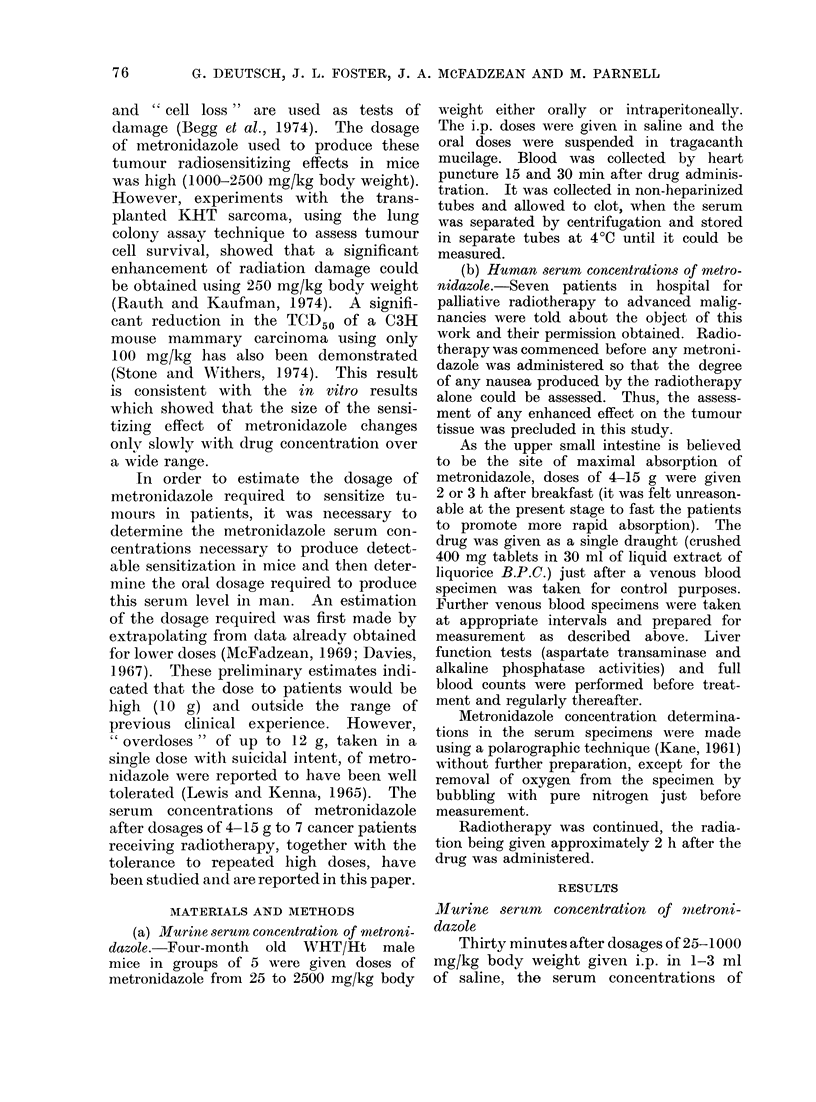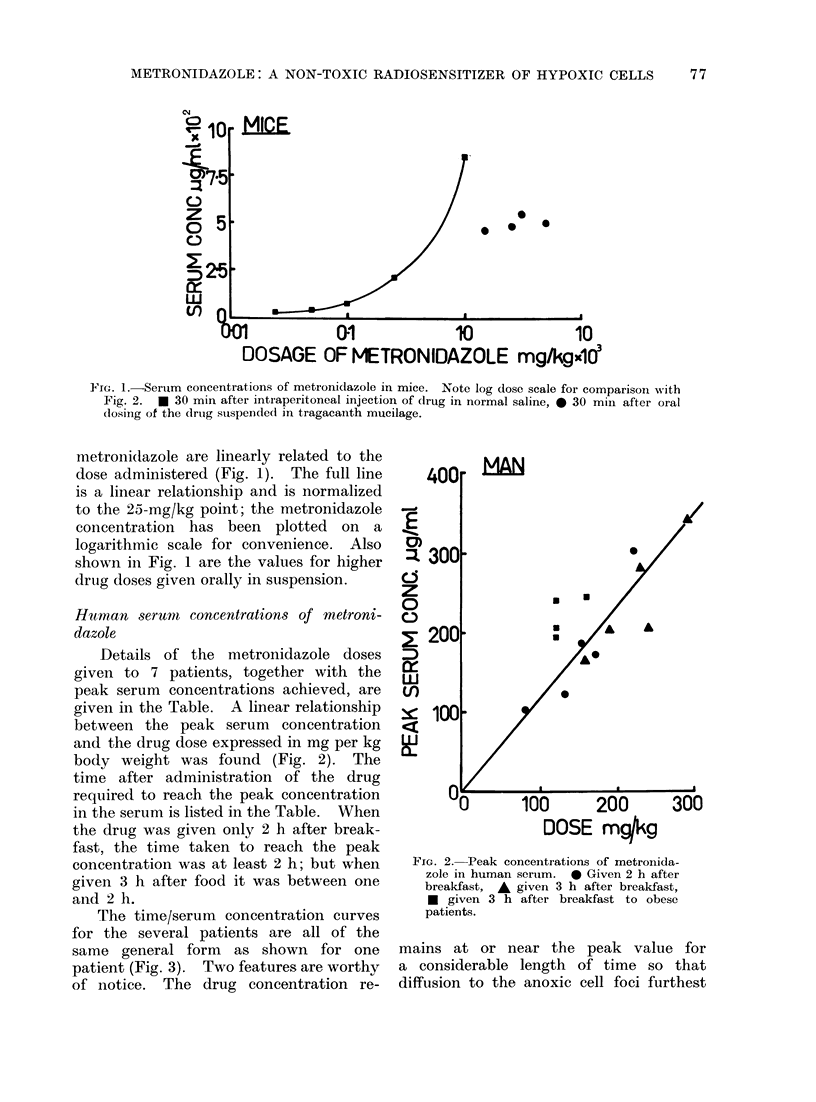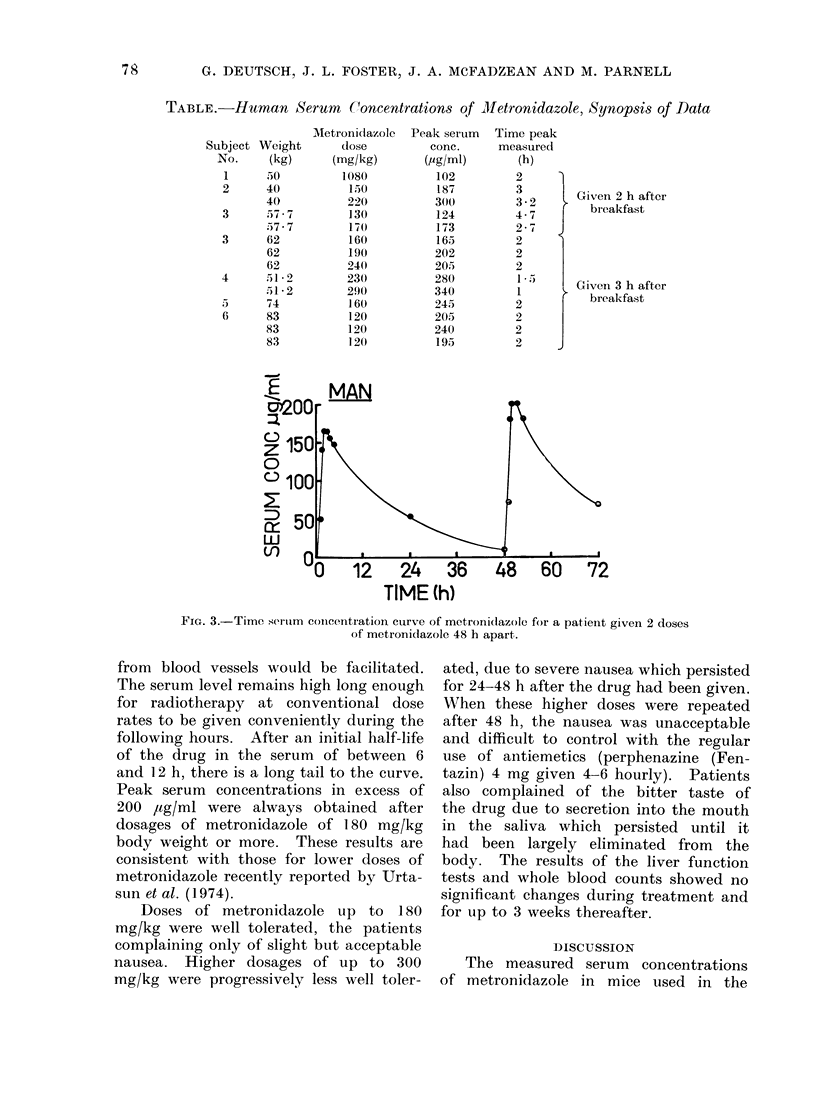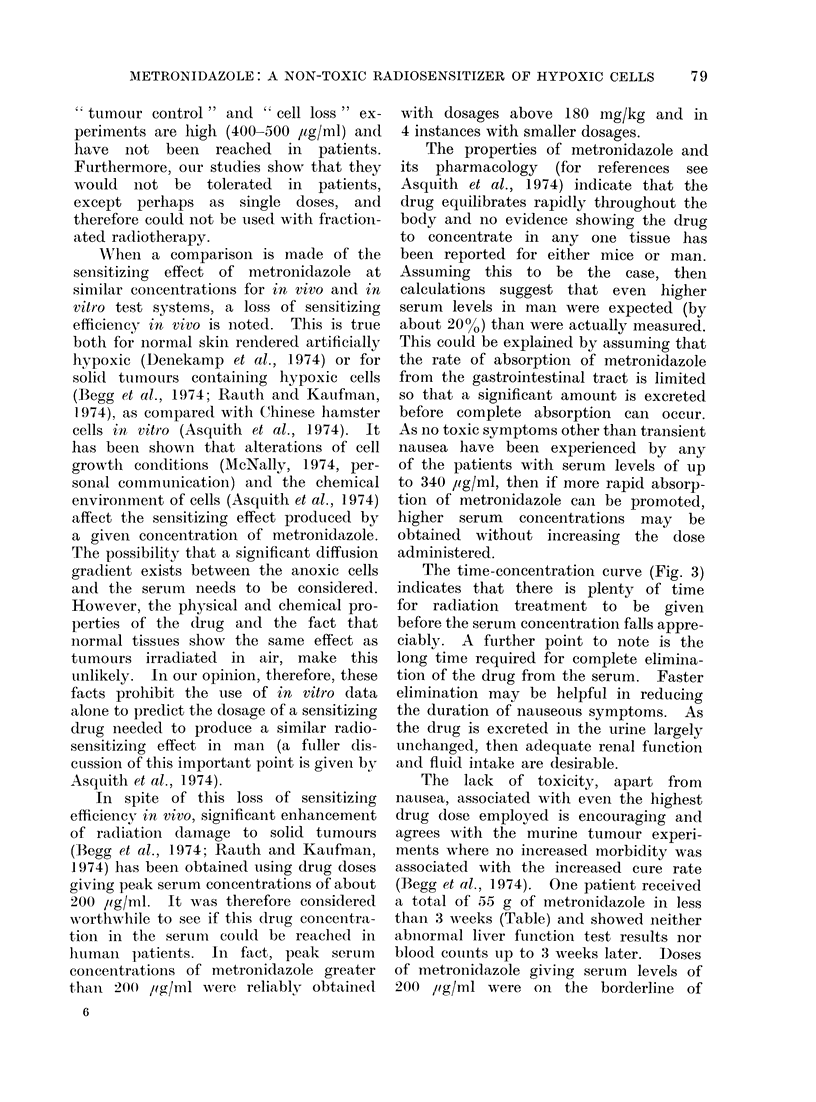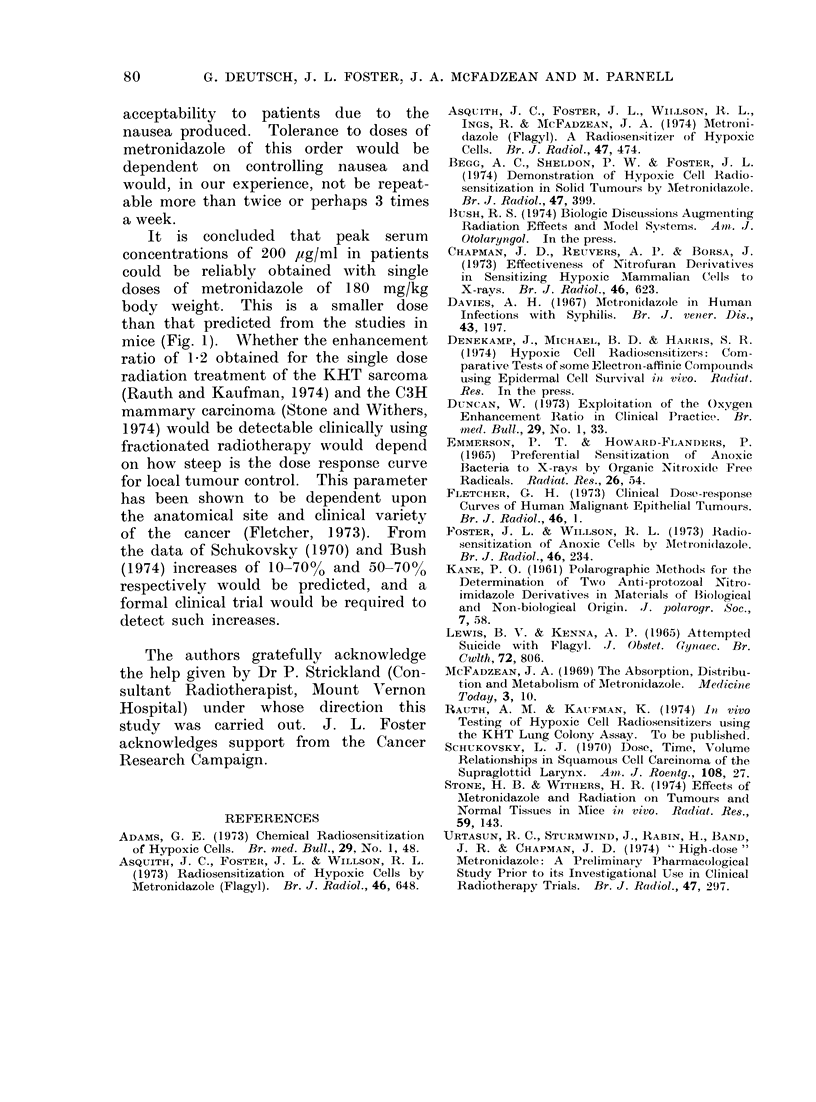# Human studies with "high dose" metronidazole: a non-toxic radiosensitizer of hypoxic cells.

**DOI:** 10.1038/bjc.1975.9

**Published:** 1975-01

**Authors:** G. Deutsch, J. L. Foster, J. A. McFadzean, M. Parnell

## Abstract

The serum concentrations of the radiosensitizer metronidazole have been determined in mice for both oral and intraperitoneal doses of the drug and these have been related to radiosensitization studies in murine tumour systems. In preliminary work before a possible clinical trail the serum metronidazole concentration/time curves have been determined in 7 patients using single doses of metronidazole of up to 15 g. The data suggest that a linear relationship exists between the metronidazole dose expressed in mg/kg and the peak serum concentration. The possibility of achieving radiosensitization of tumours in patients after tolerable doses of metronidazole is discussed in relation to enhancement ratios determined for in vitro and in vivo systems. It is concluded that predictions from in vitro systems give values that are probably too optimistic.


Br. J. Cancer (1975) 31, 75

### HUMAN STUDIES WITH "HIGH DOSE" METRONIDAZOLE: A NON-TOXIC RADIOSENSITIZER OF HYPOXIC CELLS

*G. DEUTSCH, †J. L. FOSTER, ‡J. A. McFADZEAN AND M. PARNELL

*From the *Department of Radiotherapy and †Gray Laboratory of the Cancer Research Campaign, Mount Vernon Hospital, Northwood, Middlesex, HA6 2RN and the ‡Research Laboratories, May & Baker Ltd, Dagenham, Essex, RM10 7XS*

Received 29 August 1974. Accepted 20 September 1974

**Summary.—The serum concentrations of the radiosensitizer metronidazole have been determined in mice for both oral and intraperitoneal doses of the drug and these have been related to radiosensitization studies in murine tumour systems.**

**In preliminary work before a possible clinical trial the serum metronidazole concentration/time curves have been determined in 7 patients using single doses of metronidazole of up to 15 g. The data suggest that a linear relationship exists between the metronidazole dose expressed in mg/kg and the peak serum concentration.**

**The possibility of achieving radiosensitization of tumours in patients after tolerable doses of metronidazole is discussed in relation to enhancement ratios determined for *in vitro* and *in vivo* systems. It is concluded that predictions from *in vitro* systems give values that are probably too optimistic.**

SEVERAL methods have been proposed to reduce the problem caused by hypoxic, radioresistant tumour cells in the eradication of solid human tumours by radiotherapy (Duncan, 1973). One method is to sensitize selectively the hypoxic cells using drugs (Emmerson and Howard-Flanders, 1965). Many drugs have been identified which sensitize only hypoxic cells to the lethal effects of radiation and this property has been associated with high electron affinity in these compounds (Adams, 1973). The discovery that metronidazole (Flagyl, May and Baker Ltd) selectively sensitized anoxic bacteria and bean roots (Foster and Willson, 1973), mammalian cells *in vitro* (Asquith, Foster and Willson, 1973; Chapman, Reuvers and Borsa, 1973; Asquith *et al.*, 1974) and has a favourable pharmacology and toxicology in man, led to experiments to test the radiosensitizing efficiency of this drug in animal tumours and normal tissues in experimental animals.

No radiosensitization of normal skin in oxygen breathing mice was noted using metronidazole doses of up to 3 g/kg (Denekamp, Michael and Harris, 1974). Metronidazole has been shown to reduce the dose needed to cure 50% of C3H mice carrying first generation mammary tumour transplants from 4040 rad for oxygen breathing controls to 3100 rad for oxygen breathing metronidazole treated mice (ER§ $1{\cdot}3 \pm 0{\cdot}16$). A dose of 3400 rad increases the cure rate from 20% in control mice to 70% in the drug treated mice (Begg, Sheldon and Foster, 1974).

Metronidazole has also been shown to enhance radiation induced damage to a fast growing anaplastic sarcoma and first generation transplants of C3H mammary tumour when "time to regrow"

* Present address: Royal Sussex County Hospital, Radiotherapy Department, Brighton, BN2 5BE.

† Address for reprint requests.

§ ER = Enhancement ratio; the ratio of the x-ray dose without sensitizer to that required with sensitizer to produce the same effect.

and " cell loss " are used as tests of damage (Begg *et al.*, 1974). The dosage of metronidazole used to produce these tumour radiosensitizing effects in mice was high (1000–2500 mg/kg body weight). However, experiments with the transplanted KHT sarcoma, using the lung colony assay technique to assess tumour cell survival, showed that a significant enhancement of radiation damage could be obtained using 250 mg/kg body weight (Rauth and Kaufman, 1974). A significant reduction in the $TCD_{50}$ of a C3H mouse mammary carcinoma using only 100 mg/kg has also been demonstrated (Stone and Withers, 1974). This result is consistent with the *in vitro* results which showed that the size of the sensitizing effect of metronidazole changes only slowly with drug concentration over a wide range.

In order to estimate the dosage of metronidazole required to sensitize tumours in patients, it was necessary to determine the metronidazole serum concentrations necessary to produce detectable sensitization in mice and then determine the oral dosage required to produce this serum level in man. An estimation of the dosage required was first made by extrapolating from data already obtained for lower doses (McFadzean, 1969; Davies, 1967). These preliminary estimates indicated that the dose to patients would be high (10 g) and outside the range of previous clinical experience. However, " overdoses " of up to 12 g, taken in a single dose with suicidal intent, of metronidazole were reported to have been well tolerated (Lewis and Kenna, 1965). The serum concentrations of metronidazole after dosages of 4–15 g to 7 cancer patients receiving radiotherapy, together with the tolerance to repeated high doses, have been studied and are reported in this paper.

### MATERIALS AND METHODS

(a) *Murine serum concentration of metronidazole.*—Four-month old WHT/Ht male mice in groups of 5 were given doses of metronidazole from 25 to 2500 mg/kg body weight either orally or intraperitoneally. The i.p. doses were given in saline and the oral doses were suspended in tragacanth mucilage. Blood was collected by heart puncture 15 and 30 min after drug administration. It was collected in non-heparinized tubes and allowed to clot, when the serum was separated by centrifugation and stored in separate tubes at 4°C until it could be measured.

(b) *Human serum concentrations of metronidazole.*—Seven patients in hospital for palliative radiotherapy to advanced malignancies were told about the object of this work and their permission obtained. Radiotherapy was commenced before any metronidazole was administered so that the degree of any nausea produced by the radiotherapy alone could be assessed. Thus, the assessment of any enhanced effect on the tumour tissue was precluded in this study.

As the upper small intestine is believed to be the site of maximal absorption of metronidazole, doses of 4–15 g were given 2 or 3 h after breakfast (it was felt unreasonable at the present stage to fast the patients to promote more rapid absorption). The drug was given as a single draught (crushed 400 mg tablets in 30 ml of liquid extract of liquorice *B.P.C.*) just after a venous blood specimen was taken for control purposes. Further venous blood specimens were taken at appropriate intervals and prepared for measurement as described above. Liver function tests (aspartate transaminase and alkaline phosphatase activities) and full blood counts were performed before treatment and regularly thereafter.

Metronidazole concentration determinations in the serum specimens were made using a polarographic technique (Kane, 1961) without further preparation, except for the removal of oxygen from the specimen by bubbling with pure nitrogen just before measurement.

Radiotherapy was continued, the radiation being given approximately 2 h after the drug was administered.

### RESULTS

### *Murine serum concentration of metronidazole*

Thirty minutes after dosages of 25–1000 mg/kg body weight given i.p. in 1–3 ml of saline, the serum concentrations of

FIG. 1.—Serum concentrations of metronidazole in mice. Note log dose scale for comparison with Fig. 2. ■ 30 min after intraperitoneal injection of drug in normal saline, ● 30 min after oral dosing of the drug suspended in tragacanth mucilage.

metronidazole are linearly related to the dose administered (Fig. 1). The full line is a linear relationship and is normalized to the 25-mg/kg point; the metronidazole concentration has been plotted on a logarithmic scale for convenience. Also shown in Fig. 1 are the values for higher drug doses given orally in suspension.

*Human serum concentrations of metronidazole*

Details of the metronidazole doses given to 7 patients, together with the peak serum concentrations achieved, are given in the Table. A linear relationship between the peak serum concentration and the drug dose expressed in mg per kg body weight was found (Fig. 2). The time after administration of the drug required to reach the peak concentration in the serum is listed in the Table. When the drug was given only 2 h after breakfast, the time taken to reach the peak concentration was at least 2 h; but when given 3 h after food it was between one and 2 h.

The time/serum concentration curves for the several patients are all of the same general form as shown for one patient (Fig. 3). Two features are worthy of notice. The drug concentration remains at or near the peak value for a considerable length of time so that diffusion to the anoxic cell foci furthest

FIG. 2.—Peak concentrations of metronidazole in human serum. ● Given 2 h after breakfast, ▲ given 3 h after breakfast, ■ given 3 h after breakfast to obese patients.

TABLE.—*Human Serum Concentrations of Metronidazole, Synopsis of Data*

| Subject No. | Weight (kg) | Metronidazole dose (mg/kg) | Peak serum conc. (μg/ml) | Time peak measured (h) | |
|---|---|---|---|---|---|
| 1 | 50 | 1080 | 102 | 2 | Given 2 h after breakfast |
| 2 | 40 | 150 | 187 | 3 | |
| | 40 | 220 | 300 | 3·2 | |
| 3 | 57·7 | 130 | 124 | 4·7 | |
| | 57·7 | 170 | 173 | 2·7 | |
| 3 | 62 | 160 | 165 | 2 | Given 3 h after breakfast |
| | 62 | 190 | 202 | 2 | |
| | 62 | 240 | 205 | 2 | |
| 4 | 51·2 | 230 | 280 | 1·5 | |
| | 51·2 | 290 | 340 | 1 | |
| 5 | 74 | 160 | 245 | 2 | |
| 6 | 83 | 120 | 205 | 2 | |
| | 83 | 120 | 240 | 2 | |
| | 83 | 120 | 195 | 2 | |

FIG. 3.—Time serum concentration curve of metronidazole for a patient given 2 doses of metronidazole 48 h apart.

from blood vessels would be facilitated. The serum level remains high long enough for radiotherapy at conventional dose rates to be given conveniently during the following hours. After an initial half-life of the drug in the serum of between 6 and 12 h, there is a long tail to the curve. Peak serum concentrations in excess of 200 μg/ml were always obtained after dosages of metronidazole of 180 mg/kg body weight or more. These results are consistent with those for lower doses of metronidazole recently reported by Urtasun *et al.* (1974).

Doses of metronidazole up to 180 mg/kg were well tolerated, the patients complaining only of slight but acceptable nausea. Higher dosages of up to 300 mg/kg were progressively less well tolerated, due to severe nausea which persisted for 24–48 h after the drug had been given. When these higher doses were repeated after 48 h, the nausea was unacceptable and difficult to control with the regular use of antiemetics (perphenazine (Fentazin) 4 mg given 4–6 hourly). Patients also complained of the bitter taste of the drug due to secretion into the mouth in the saliva which persisted until it had been largely eliminated from the body. The results of the liver function tests and whole blood counts showed no significant changes during treatment and for up to 3 weeks thereafter.

### DISCUSSION

The measured serum concentrations of metronidazole in mice used in the

"tumour control" and "cell loss" experiments are high (400–500 $\mu$g/ml) and have not been reached in patients. Furthermore, our studies show that they would not be tolerated in patients, except perhaps as single doses, and therefore could not be used with fractionated radiotherapy.

When a comparison is made of the sensitizing effect of metronidazole at similar concentrations for *in vivo* and *in vitro* test systems, a loss of sensitizing efficiency *in vivo* is noted. This is true both for normal skin rendered artificially hypoxic (Denekamp *et al.*, 1974) or for solid tumours containing hypoxic cells (Begg *et al.*, 1974; Rauth and Kaufman, 1974), as compared with Chinese hamster cells *in vitro* (Asquith *et al.*, 1974). It has been shown that alterations of cell growth conditions (McNally, 1974, personal communication) and the chemical environment of cells (Asquith *et al.*, 1974) affect the sensitizing effect produced by a given concentration of metronidazole. The possibility that a significant diffusion gradient exists between the anoxic cells and the serum needs to be considered. However, the physical and chemical properties of the drug and the fact that normal tissues show the same effect as tumours irradiated in air, make this unlikely. In our opinion, therefore, these facts prohibit the use of *in vitro* data alone to predict the dosage of a sensitizing drug needed to produce a similar radiosensitizing effect in man (a fuller discussion of this important point is given by Asquith *et al.*, 1974).

In spite of this loss of sensitizing efficiency *in vivo*, significant enhancement of radiation damage to solid tumours (Begg *et al.*, 1974; Rauth and Kaufman, 1974) has been obtained using drug doses giving peak serum concentrations of about 200 $\mu$g/ml. It was therefore considered worthwhile to see if this drug concentration in the serum could be reached in human patients. In fact, peak serum concentrations of metronidazole greater than 200 $\mu$g/ml were reliably obtained with dosages above 180 mg/kg and in 4 instances with smaller dosages.

The properties of metronidazole and its pharmacology (for references see Asquith *et al.*, 1974) indicate that the drug equilibrates rapidly throughout the body and no evidence showing the drug to concentrate in any one tissue has been reported for either mice or man. Assuming this to be the case, then calculations suggest that even higher serum levels in man were expected (by about 20%) than were actually measured. This could be explained by assuming that the rate of absorption of metronidazole from the gastrointestinal tract is limited so that a significant amount is excreted before complete absorption can occur. As no toxic symptoms other than transient nausea have been experienced by any of the patients with serum levels of up to 340 $\mu$g/ml, then if more rapid absorption of metronidazole can be promoted, higher serum concentrations may be obtained without increasing the dose administered.

The time-concentration curve (Fig. 3) indicates that there is plenty of time for radiation treatment to be given before the serum concentration falls appreciably. A further point to note is the long time required for complete elimination of the drug from the serum. Faster elimination may be helpful in reducing the duration of nauseous symptoms. As the drug is excreted in the urine largely unchanged, then adequate renal function and fluid intake are desirable.

The lack of toxicity, apart from nausea, associated with even the highest drug dose employed is encouraging and agrees with the murine tumour experiments where no increased morbidity was associated with the increased cure rate (Begg *et al.*, 1974). One patient received a total of 55 g of metronidazole in less than 3 weeks (Table) and showed neither abnormal liver function test results nor blood counts up to 3 weeks later. Doses of metronidazole giving serum levels of 200 $\mu$g/ml were on the borderline of acceptability to patients due to the nausea produced. Tolerance to doses of metronidazole of this order would be dependent on controlling nausea and would, in our experience, not be repeatable more than twice or perhaps 3 times a week.

It is concluded that peak serum concentrations of 200 μg/ml in patients could be reliably obtained with single doses of metronidazole of 180 mg/kg body weight. This is a smaller dose than that predicted from the studies in mice (Fig. 1). Whether the enhancement ratio of 1·2 obtained for the single dose radiation treatment of the KHT sarcoma (Rauth and Kaufman, 1974) and the C3H mammary carcinoma (Stone and Withers, 1974) would be detectable clinically using fractionated radiotherapy would depend on how steep is the dose response curve for local tumour control. This parameter has been shown to be dependent upon the anatomical site and clinical variety of the cancer (Fletcher, 1973). From the data of Schukovsky (1970) and Bush (1974) increases of 10–70% and 50–70% respectively would be predicted, and a formal clinical trial would be required to detect such increases.

The authors gratefully acknowledge the help given by Dr P. Strickland (Consultant Radiotherapist, Mount Vernon Hospital) under whose direction this study was carried out. J. L. Foster acknowledges support from the Cancer Research Campaign.

### REFERENCES

ADAMS, G. E. (1973) Chemical Radiosensitization of Hypoxic Cells. *Br. med. Bull.*, **29**, No. 1, 48.

ASQUITH, J. C., FOSTER, J. L. & WILLSON, R. L. (1973) Radiosensitization of Hypoxic Cells by Metronidazole (Flagyl). *Br. J. Radiol.*, **46**, 648.

ASQUITH, J. C., FOSTER, J. L., WILLSON, R. L., INGS, R. & MCFADZEAN, J. A. (1974) Metronidazole (Flagyl). A Radiosensitizer of Hypoxic Cells. *Br. J. Radiol.*, **47**, 474.

BEGG, A. C., SHELDON, P. W. & FOSTER, J. L. (1974) Demonstration of Hypoxic Cell Radiosensitization in Solid Tumours by Metronidazole. *Br. J. Radiol.*, **47**, 399.

BUSH, R. S. (1974) Biologic Discussions Augmenting Radiation Effects and Model Systems. *Am. J. Otolaryngol.* In the press.

CHAPMAN, J. D., REUVERS, A. P. & BORSA, J. (1973) Effectiveness of Nitrofuran Derivatives in Sensitizing Hypoxic Mammalian Cells to X-rays. *Br. J. Radiol.*, **46**, 623.

DAVIES, A. H. (1967) Metronidazole in Human Infections with Syphilis. *Br. J. vener. Dis.*, **43**, 197.

DENEKAMP, J., MICHAEL, B. D. & HARRIS, S. R. (1974) Hypoxic Cell Radiosensitizers: Comparative Tests of some Electron-affinic Compounds using Epidermal Cell Survival *in vivo*. *Radiat. Res.* In the press.

DUNCAN, W. (1973) Exploitation of the Oxygen Enhancement Ratio in Clinical Practice. *Br. med. Bull.*, **29**, No. 1, 33.

EMMERSON, P. T. & HOWARD-FLANDERS, P. (1965) Preferential Sensitization of Anoxic Bacteria to X-rays by Organic Nitroxide Free Radicals. *Radiat. Res.*, **26**, 54.

FLETCHER, G. H. (1973) Clinical Dose-response Curves of Human Malignant Epithelial Tumours. *Br. J. Radiol.*, **46**, 1.

FOSTER, J. L. & WILLSON, R. L. (1973) Radiosensitization of Anoxic Cells by Metronidazole. *Br. J. Radiol.*, **46**, 234.

KANE, P. O. (1961) Polarographic Methods for the Determination of Two Anti-protozoal Nitroimidazole Derivatives in Materials of Biological and Non-biological Origin. *J. polarogr. Soc.*, **7**, 58.

LEWIS, B. V. & KENNA, A. P. (1965) Attempted Suicide with Flagyl. *J. Obstet. Gynaec. Br. Cwlth*, **72**, 806.

MCFADZEAN, J. A. (1969) The Absorption, Distribution and Metabolism of Metronidazole. *Medicine Today*, **3**, 10.

RAUTH, A. M. & KAUFMAN, K. (1974) *In vivo* Testing of Hypoxic Cell Radiosensitizers using the KHT Lung Colony Assay. To be published.

SCHUKOVSKY, L. J. (1970) Dose, Time, Volume Relationships in Squamous Cell Carcinoma of the Supraglottid Larynx. *Am. J. Roentg.*, **108**, 27.

STONE, H. B. & WITHERS, H. R. (1974) Effects of Metronidazole and Radiation on Tumours and Normal Tissues in Mice *in vivo*. *Radiat. Res.*, **59**, 143.

URTASUN, R. C., STURMWIND, J., RABIN, H., BAND, J. R. & CHAPMAN, J. D. (1974) "High-dose" Metronidazole: A Preliminary Pharmacological Study Prior to its Investigational Use in Clinical Radiotherapy Trials. *Br. J. Radiol.*, **47**, 297.